# Antioxidant/Prooxidant and Antibacterial/Probacterial Effects of a Grape Seed Extract in Complex with Lipoxygenase

**DOI:** 10.1155/2014/313684

**Published:** 2014-09-15

**Authors:** Veronica Sanda Chedea, Cornelia Braicu, Flore Chirilă, Henry Joseph Oduor Ogola, Rodica Ştefania Pelmuş, Loredana Georgeta Călin, Carmen Socaciu

**Affiliations:** ^1^Laboratory of Animal Biology, National Research Development Institute for Animal Biology and Nutrition (IBNA), Calea Bucureşti nr. 1, Baloteşti, 077015 Ilfov, Romania; ^2^Research Center for Functional Genomics, Biomedicine and Translational Medicine, “Iuliu Hatieganu” University of Medicine and Pharmacy, 400 565 Cluj-Napoca, Romania; ^3^Department of Microbiology-Immunology, University of Agricultural Sciences and Veterinary Medicine, 3-5 Manastur Street, 400372 Cluj-Napoca, Romania; ^4^School of Agricultural and Food Sciences, Jaramogi Oginga Odinga University of Science and Technology, P.O. Box 210, Bondo 40601, Kenya; ^5^Department of Chemistry and Biochemistry, University of Agricultural Sciences and Veterinary Medicine, 3-5 Manastur Street, 400372 Cluj-Napoca, Romania

## Abstract

In an attempt to determine the antioxidant/prooxidant, antibacterial/probacterial action of flavan-3-ols and procyanidins from grape seeds, pure catechin (CS), and an aqueous grape seed extract (PE), were applied in the absence and presence of pure lipoxygenase (LS) or in extract (LE) to leucocyte culture, *Escherichia coli* *B*_41_ and *Brevibacterium linens*, and observed whether there was any effect on lipid peroxidation, cytotoxicity, or growth rate. Short time periods of coincubation of cells with the polyphenols, followed by the exposure to LS and LE, revealed a high level of lipid peroxidation and a prooxidative effect. Longer coincubation and addition of LS and LE resulted in the reversal of the prooxidant action either to antioxidant activity for CS + LS and PE + LS or to the control level for CS + LE and PE + LE. Lipid peroxidation was significantly reduced when cells were exposed to polyphenols over a longer period. Longer exposure of *E. coli* to CS or PE followed by addition of LS for 3 h resulted in bactericidal activity. Significant stimulatory effect on microbial growth was observed for PE + LS and PE + LE treatments in *B. linens*, illustrating the potential probacterial activity in *B. linens* cultures. Lipoxygenase-polyphenols complex formation was found to be responsible for the observed effects.

## 1. Introduction

During grape processing, it is estimated that 20% of total weight of grape fruits used results in grape pomace that presents a challenging waste disposal problem for the winery and grape juice industry [1]. Winemaking by-products are of particular interest because grape is the world's largest fruit crop, with more than 68 million tons produced per year. The European Union affords approximately 24.5 million tons per year, and Romania produces 740.118 tons of grapes ranking the 19th place in the world (in 2010). It also produces 125.450 tons of wine [2]. Therefore, solutions involving further processing of the grape pomace to provide useful products that may balance out waste treatment costs are very important [3]. An alternative utilisation of the grape pomace could involve the isolation of the grape seeds and extraction of the polyphenols. Among the total extractable phenolics from grapes, approximately 60–70% comprises catechins, epicatechins, procyanidins, and proanthocyanidins, a group of important polyphenols that exert a beneficial effect on human health [4–6]. Grape seeds contain lipid, protein, carbohydrates, and 5–8% polyphenols, depending on the variety. The most abundant phenolic compounds isolated from grape seeds are catechin, epicatechin, and procyanidins [4]. Grape seed proanthocyanidins constitute a complex mixture that consisted of procyanidins and procyanidin gallates [5].

Grape seed extract (GSE) has been reported to possess a broad spectrum of pharmacological and therapeutic effects including anti-inflammatory activity and can reduce apoptotic cell death [7, 8]. The proanthocyanidins from GSE have shown promising chemopreventive and/or anticancer properties in various cell culture and animal models [9]. The findings of Feng et al. [10] indicate that grape seed extract has neuroprotective properties in the neonatal rat hypoxia-ischemic brain injury model. The results also indicate that the suppression of free radicals after hypoxic ischemia by grape seed extract is one potential mechanism of this neuroprotection. Oxidative stress, the consequence of an imbalance of prooxidants and antioxidants in the organism, has rapidly gained recognition as a key phenomenon in chronic diseases: cardiovascular disease, hypertension, diabetes mellitus, and cancer [11]. The harmful effects of oxidative processes in living organisms can be reduced by the dietary intake of flavan-3-ols and procyanidins [12].

Lipoxygenase (LOX, EC 1.13.11.12), a dioxygenase known to be widely distributed in plants, animals, and microorganisms, catalyses the oxidation of polyunsaturated fatty acids to hydroperoxides [13]. Peroxyl radical complexes have been reported to exist during the catalytic cycle of LOX and can serve as sources of free radicals [14]. Thus, lipoxygenase can be seen as an oxidative stress inducer and also oxidative stress may favor a concerted package of lipoxygenase-mediated enzymatic and no-enzymatic lipid peroxidation and cooxidative processes [11]. Considering the various detrimental effects of imbalances or perturbations in fatty acid oxidation, a considerable interest in the development and characterization of LOX inhibitors was reported [15, 16]. Antioxidants such as flavonoids, which act as free radical quenchers, may act also as LOX inhibitors [17]. Schewe et al. [18, 19] studying the inhibitory effect of (−) epicatechin and of related oligomers, procyanidins, towards mammalian lipoxygenase, suggest that the general lipoxygenase inhibitory potency of flavanols and procyanidins may contribute to their beneficial effects on the cardiovascular system in man.

Phenolic compounds from grape seeds have pharmacological and nutraceutical benefits showing antiviral and antimutagenic actions [20] that are closely related to their antioxidant and singlet oxygen quenching ability. Recognition of such health benefits of catechins and procyanidins has led to the use of grape seed extract as a dietary supplement [21, 22]. Besides its antioxidant activity, the grape seed extract proved to act also as antibacterial agent [23–25].

In an attempt to determine the beneficial properties for the human health of the flavan-3-ols and procyanidins from grape seeds—through food or immediate house medical care—and also the exploitation of the potential added-value of this by-product, the effects of pure catechin and of an aqueous grape seed extract was assessed in this study. The evaluation was done in the absence and presence of pure lipoxygenase or in extract, on the lipid peroxidation and cytotoxicity on leucocyte culture as well as on the growth rate of* Brevibacterium linens* and* Escherichia coli B*_41_.

*E. coli *is the most common cause of infections by Gram-negative bacilli and the bacterial organism most often isolated from blood cultures. It is a frequent cause of outpatient urinary tract infections in women worldwide, of hospitalization due to pyelonephritis and septicemia, and of nosocomial infections among hospitalized patients. Meningitis caused by* E. coli *in neonates is frequently fatal. Resistance to recommended first- and second-line agents, such as penicillins, cephalosporins, sulfa drugs, and fluoroquinolones, is high in many countries and is commonly associated with treatment failure [26]. For observing and comparing the differences on Gram-positive bacteria,* B. linens* was used in this study.

Based on previous studies [25, 27] using the UV-Vis spectroscopy, interactions between LOX soybean extract and the catechin-type compounds from grape seed extract in leukocyte and bacterial culture were monitored addressing the issue of possible inhibition of lipoxygenase by catechins as well as the complexion of the enzyme with these polyphenols in inducing an antioxidant/prooxidant or antibiotic/probacterial action.

As a prooxidant inducer, the standard soybean lipoxygenase as well as a raw extract from soybean containing LOX-1 and LOX-3 isoenzymes were used to determine potential inhibitory activity of different classes of polyphenols towards LOX enzymes [28]. Flavonoids, in particular those containing a catechol group, are known to chelate iron and other transition metal ions, and lipoxygenases contain an iron moiety at the active site [29]. This possible chelation through a prooxidant/antioxidant effect was evaluated by the TBARS (thiobarbituric acid reactive substances**) **and MTT assays on the leucocyte culture.

Lipid peroxidation is a classic indicator of oxidative stress whereby free radicals extract electrons from cell membranes inflicting damage. Quantification of lipid peroxidation uses malondialdehyde (MDA), the end product of lipid peroxidation, and uses the reaction between MDA and thiobarbituric acid (TBA), which yields thiobarbituric acid reactive substances (TBARS) which can be quantified by visible or fluorescence spectrophotometry [30]. Although the thiobarbituric acid assay is not specific for MDA and several other aldehydic products of cellular molecules can react with TBA, it is the most commonly used method to determine lipid peroxidation [10].

## 2. Materials and Methods

### 2.1. Chemicals

Catechin standard ((±)-catechin hydrate) and pure soybean lipoxygenase-1 were purchased from Sigma Chemical Co., St. Louis, MO. The standard enzyme contained 46.000 units/mg protein. Other chemicals used were all analytical grade. RPMI medium, fetal serum, penicillin, streptomycin, L-glutamine, Triton, Hanks salt containing MTT ((3-(4,5-dimethylthiazolyl-2)-2,5-diphenyltetrazolium bromide), and DMSO (dimethyl sulfoxide) were from Sigma, Cluj-Napoca, Romania.

### 2.2. Microbial Strains

Catechin standard (CS), LOX-1 standard (LS), and two extracts (grape seed polyphenolic extract (PE) and LOX soybean extract (LE) were tested against* Escherichia coli B*_41_-a reference strain and* Brevibacterium linens*.* Escherichia coli B*_41_ was obtained from the culture collection of microbial strains of the Department of Microbiology, Faculty of Veterinary Medicine, Cluj-Napoca, Romania.* Brevibacterium linens* was isolated from the fermented cheese Năsal, a Romanian brand of maturated cheese, and maintained in the same collection as above.

Bacterial strains cultured overnight at 37°C in agar were transferred into 250 mL peptone broth (Difco Laboratories) kept in a reciprocal shaker at 37°C for 24 h yielding a stock preparation with a log-phase cell density of approximately 10^7^ colony forming units (CFU)/mL as evaluated initially by measurements of the optical density at 630 nm.

### 2.3. Standard Solutions Preparation (CS and LS)

Catechin was solubilized in pure Milli-Q water (CS). CS was then added to the cell or microbial culture to a final concentration of 310 *μ*M. Lipoxygenase standard was solubilized in physiological saline buffer (PBS) pH = 7, as a stock solution of 1 mg/mL protein corresponding to 46.000 enzymatic units/mL in PBS (LS). In the experiments, 575 e.u. LOX/mL leucocyte or bacterial culture medium was used.

### 2.4. Extraction of Polyphenols from Grape Seeds (PE) and LOX from Soybeans (LE)

The polyphenols from grape seeds were extracted and characterized as previously described [31]. The total content of the catechin type compounds in this extract as determined by HPLC was 1956 mg total catechins/Kg dry grape seeds [31].

To obtain LE, an aliquot of 5 g soybean meal was mixed with 30 mL of PBS, pH 7.0, stirred for 1 h at room temperature, filtered through cheesecloth, and then centrifuged at 16,000 rpm for 10 min. The resultant supernatant of the raw extract was designated as the lipoxygenase extract, LE. The total protein content of LE, determined as described by Gornall et al. [32], was 27 *μ*g total protein/mL extract.

### 2.5. Total Polyphenol Content of PE and LE

The total polyphenol content measured by the Folin-Ciocalteu method [33] was 3.2 g gallic acid equivalents (GAE)/kg grape seeds and 1.8 g gallic acid equivalents/kg soybean.

### 2.6. Leukocytes Isolation and Treatment

Viable leukocytes were obtained from sterile, whole horse blood drawn with plastic syringe containing cold heparin solution in physiological saline buffer at final concentration of 50 UI/mL. Once drawn, gravitational sedimentation of erythrocytes was performed by centrifugation for 10 min at 1500 rpm, (blood is separated into plasma or “buffy coat” phase of leukocytes above the erythrocyte phase). The plasma phase was then transferred to a fresh tube and centrifuged again for 10 min at 3500 rpm. The supernatant was discarded and the pellet was washed with PBS. After a further centrifugation of 10 min at 3500 rpm, the pellet was suspended in media to a concentration of 1 × 10^6^ cells/mL. Leukocytes were cultured in RPMI medium supplemented with 10% fetal serum, 100 U/mL penicillin, 100 mg/mL streptomycin, and 2 mM L-glutamine under standard culture condition (37°C, 95% humidified air and 5% CO_2_) [27].

Then, 36 *μ*L of CS or PE was added on the cell culture. Two incubation protocols were used for the polyphenols and leucocyte: 3 h (short incubation time) and 24 h (long incubation time). After incubation, 250 *μ*L of LS or LE was added and incubated for 1 hour before TBARS and MTT assays were performed.

### 2.7. Determination of Thiobarbituric Acid Reactive Substances (TBARS Assay)

**Figure 1:**
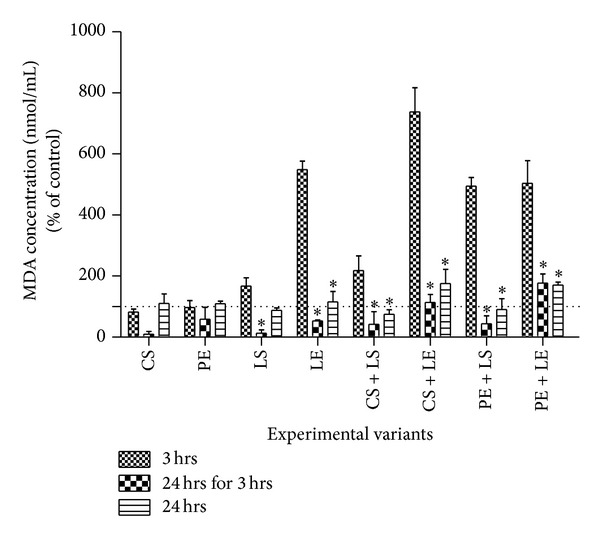
Effect of grape seed extract on leucocytes thiobarbituric acid reactive substances (TBARS). Treatment with the pure catechin (CS to a final concentration of 310 *µ*M/mL medium) and grape seed extract (PE to a final concentration of 35 *µ*g polyphenols/mL medium) was administrated at the initiation of the culture (3 hrs and 24 hrs for 3 hrs) and at 24 h (24 hrs). Leucocyte thiobarbituric acid reactive substances were assessed 4 h (3 hrs) and 24 h (24 hrs for 3 hrs) after adding the polyphenols (CS and PE) for the same cell sample and at 25 h (24 hrs) for another one. Data are presented as mean ± S.E.M. Values are expressed as percent of control for the experimental variants tested: LS-standard lipoxygenase, LE-lipoxygenase extract, CS-catechin standard, and PE-polyphenolic extract.

The influence of the polyphenols and lipoxygenase interaction on leucocyte's lipid peroxidation was evaluated by TBARS assay, using suspension of 2 × 10^5^ cells per well in 12-well plates. Two incubation protocols were used for the polyphenols and leucocyte: 3 h (short incubation time) and 24 h (long incubation time). After incubation (3 h or 24 h) with polyphenol (CS and PE) and while in logarithmic growth cell phase, cells were treated with lipoxygenase (LS and LE). TBARS assay was then performed at 4 h (3 hrs in Figure 1) and 24 h (24 hrs for 3 hrs in Figure 1) for the experimental variant when LS and LE were added after 3 h. At 25 hours after the antioxidant treatment the assay was done for the experimental variant when LOX (LS and LE) was added after 24 h (24 hrs in Figure 1).

For determining lipid peroxidation, 1 mL of leucocyte culture homogenate was mixed with 2 mL working solution containing 15% (w/v) thiobarbituric acid, 0.25 N HCl. The mixture was heated for 15 min in boiling water. After cooling, the precipitate was removed by centrifugation at 3500 rpm for 10 min. Absorbance was determined at 535 nm using a spectrophotometer JASCO V-500, Cluj-Napoca, Romania. The MDA concentration was determined from a calibration curve. Results were expressed as the percentage (%) of MDA concentration, assuming the absorbance of control cells as 100%.

### 2.8. MTT Assay

**Figure 2:**
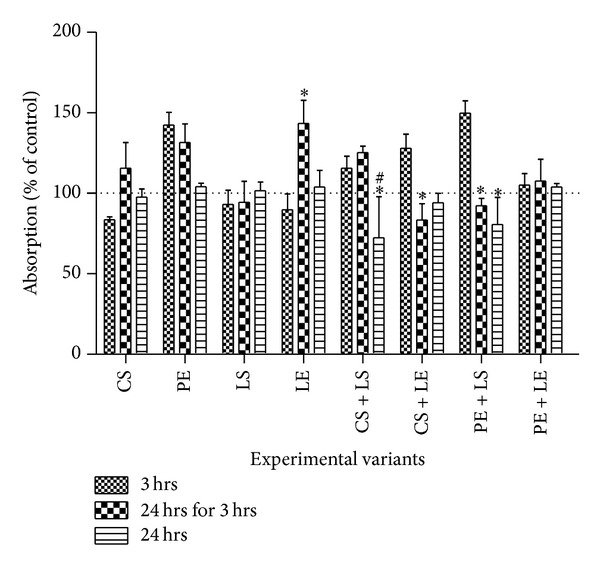
Cellular respiration (as measured by the mitochondrial MTT conversion to formazan) after coincubation with different mixtures of polyphenols (CS and PE) and lipoxygenases (LS and LE) for different periods (3 hs and 24 hs). Treatment with the pure catechin (CS to a final concentration of 310 *µ*M/mL medium) and grape seed extract (PE to a final concentration of 35 *µ*g polyphenols/mL medium) was administrated at the initiation of the culture (3 hrs and 24 hrs for 3 hrs) and at 24 h (24 hrs). Leucocyte respiration was assessed 4 h (3 hrs) and 24 h (24 hrs for 3 hrs) after adding the polyphenols (CS and PE) for the same cell sample and at 25 h (24 hrs) for another one. Data are presented as mean ± S.E.M. Values are expressed as percent of control for the experimental variants tested: LS-standard lipoxygenase, LE-lipoxygenase extract, CS-catechin standard, and PE-polyphenolic extract.

The influence of interaction between polyphenols and lipoxygenase on leucocyte's mitochondrial respiration was evaluated by MTT assay, using suspension of 2 × 10^3^ cells per well in 96-well plates. Two incubation protocols were used for the polyphenols and leucocyte: 3 h (short incubation time) and 24 h (long incubation time). After incubation with polyphenol (CS and PE), while in logarithmic growth cell phase, cells were treated with lipoxygenase (LS and LE). MTT assay was performed at 4 h (3 hrs in Figure 2) and 24 h (24 hrs for 3 hrs in Figure 2) for the experimental variant when LS and LE were added after 3 h. At 25 h after the antioxidant treatment the assay was done for the experimental variant when LOX (LS and LE) was added after 24 h (24 hrs in Figure 2).

For the MTT assay, cells were pelleted and washed with PBS and 150 *μ*L Hanks salt containing MTT (455 *μ*g/mL) was added into each well. After 2-hour incubation under standard conditions the MTT solution was removed and 200 *μ*L of DMSO was added into each well. Absorbance was measured at 490 nm using a Biotek Synergy HT Microplate Reader (Cluj-Napoca, Romania). Results were expressed as the percentage (%) of MTT reduction, assuming the absorbance of control cells as 100%.

### 2.9. Interactions between Lipoxygenase (LS or LE) and Polyphenols (CS and PE) on* E. coli* and* B. linens* Cultures: Growth Inhibition Assay (Turbidimetry and Spectrophotometry)

**Figure 3:**
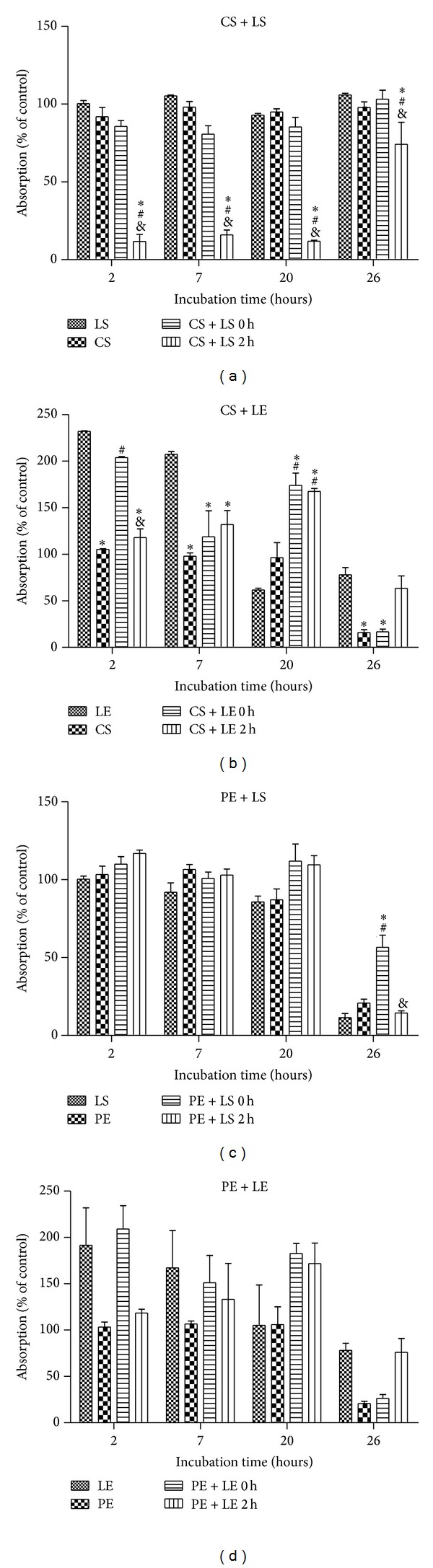
Growth rate, as referred to control, of* E. coli B*_41_ measured by UV-Vis spectroscopy at 630 nm at different time intervals (2, 7, 20, and 26 h). ∗: statistically different when compared with LS or LE, #: statistically different when compared with CS or PE, and &: statistically different when compared with CS + LS 0 h, CS + LE 0 h, PE + LS 0 h, or PE + LE 0 h for *P* < 0.05.

**Figure 4:**
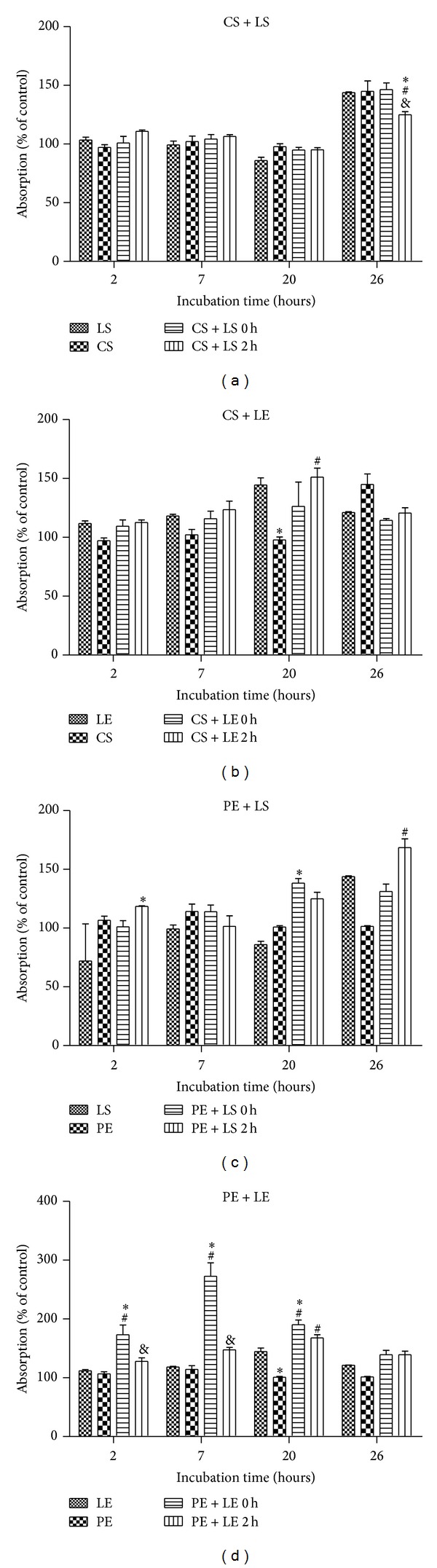
Growth rate, as referred to control, of* B. linens* measured by UV-Vis spectroscopy at 630 nm at different time intervals (2, 7, 20, and 26). ∗: statistically different when compared with LS or LE, #: statistically different when compared with CS or PE, and &: statistically different when compared with CS + LS 0 h, CS + LE 0 h, PE + LS 0 h, or PE + LE 0 h for *P* < 0.05.

Ten microliters of logarithmic-phase bacterial cultures (10^7^ CFU/mL) in 200 *μ*L nutrient broth was added in each well, in 48 wells plates. CS, to a final concentration of 310 *μ*M or 36 *μ*L and PE, to a final concentration of 35 *μ*g polyphenols/mL medium, were cocultivated with the microorganisms (Figures 3 and 4 experimental variants CS and PE). LS and LE were also incubated with bacteria, at a final concentration of 575 e.u. LOX/mL buffer for LS and 27 *μ*g total protein/mL extract for LE (Figures 3 and 4 experimental variants LS and LE). The same final concentration of LS and LE was added after 3 h to the microbial cultures treated with CS and PE (Figures 3 and 4, treatments CS + LS 3 h, CS + LE 3 h, PE + LS 3 h, and PE + LE 3 h).

In a volume of 2 mL PBS pH = 7, 36 *μ*L CS (to a final concentration of 310 *μ*M) or 36 *μ*L PE (to a final concentration of 35 *μ*g polyphenols/mL medium) was added. Each experimental variant (CS or PE in PBS) was mixed with 250 *μ*L LS diluted 1 : 10 (to a final conc. of 575 e.u. LOX/mL buffer) and also with 250 *μ*L LE (27 *μ*g total protein/mL extract). The mixture was kept for 3 h at 37°C and then added on the bacterial culture (Figures 3 and 4 experimental variants CS + LS 0 h, CS + LE 0 h, PE + LS 0 h, and PE + LE 0 h).

The cultures were incubated at 37°C for 26 h and growth inhibition was measured by determination of the absorbance at 630 nm. Absorbance readings (630 nm) were taken periodically (at 2 h, 7 h, 20 h, and 24 h).

### 2.10. Statistical Analysis

Data were presented as the mean percentages of control ± standard deviation from at least three independent experiments. Experimental data were analysed with the program Graph Pad Prism 5, performing two-way analysis of variance (ANOVA) and Bonferroni posttest was used to compare the experimental variants (a *P* < 0.05 was considered significant).

## 3. Results and Discussion

The aqueous extract of polyphenols from the grape seeds was analyzed through LC-UV-DAD and LC-ESI-MS and the quantitative analysis of its components was performed as previously reported [31]. The composition of polyphenols of the extracts tested (PE and LE) as evaluated using the LC-MS technique revealed that epicatechin and catechin were the major compounds in PE, representing together with epicatechin gallate (ECG) 60% of total polyphenols, followed by procyanidin dimers (28%) and trimers (12%) [28]. In LE, isoflavones daidzein and genistein were the main polyphenolic compounds identified, although in very low quantity consistent with our previous study [34].

### 3.1. Influence of Polyphenols (CS and PE) on Leukocytes Lipid Peroxidation (TBARS Assay) in Presence and Absence of LOX (LS or LE)

In this study, very high levels of TBARS were observed after 3-hour incubation of polyphenols and 1-hour incubation with LS or LE in the case of the experimental variants LE, CS + LE, PE + LS, and PE + LE (Figure 1). When LOXs and polyphenols were coincubated with the leucocyte culture for 21 h (24 hrs for 3 hrs), there was a significant decrease of TBARS levels lower than control with statistically significant values measured for LE, CS + LE, and PE + LS treatments. In contrast, coincubation of cells with polyphenols for 24 h followed with the addition of LS or LE for 1 h resulted in no significant antioxidant activity for any of the experimental variants tested.

In Figure 1, it can also be observed that longer incubation time (24 h versus 3 h) of polyphenols (CS or PE) correlated well with lower lipid peroxidation for LS, LE, CS + LS, CS + LE, PE + LS, and PE + LE treatments. However, the addition of LE after CS or PE elicited prooxidant activity rather any antioxidant tendency for all the incubation times tested.

### 3.2. The Effect of Polyphenols (CS and PE) on Cellular Respiration (MTT Test) in the Presence of LOX (LS or LE)

The percentage of inhibition or stimulation of respiration (measured by the MTT reduction to formazan) for cells coincubated with CS, PE with/without LS, or LE is illustrated (Figure 2). Respiration is generally stimulated under oxidative stress, if a similar number of cells are cultivated (same protein content). Consequently, high values of MTT correlate with a prooxidative activity, while lower values correspond to antioxidant action [27].

As shown in Figure 2, shorter incubation of cells with CS (3 h) was associated with antioxidant effect (experimental variant* 3 *hrs in Figure 2). However, CS incubation for 24 h (*24 *hrs* for 3 *hrs) resulted in the slight observation of prooxidant effect, although no effect was observed at 25 h (*24 *hrs). This observation is consistent with argument that longer incubation time leads to increased mitochondrial respiration due to the formation of the oxidation products [27]. MTT test also revealed that LS for all the incubation times and LE incubated for 1 h (*3 *hrs and* 24 *hrs) with the cells exhibited no significant effect on the mitochondrial respiration (Figure 2).

During coincubation of cells with the polyphenols for 3 h followed by the exposure to either LS or LE, the measured MTT after 4 h (*3 *hrs) revealed a prooxidative effect of CS + LS, CS + LE, PE + LS, and PE + LE samples. This prooxidative intensity was highest for PE + LE, while PE + LS showed the lowest intensity. In contrast, longer coincubation of cells with polyphenols for 24 h and addition of LS and LE for 1 h (*24 *hrs) prior to analysis resulted in the reversal of the prooxidant action either to antioxidant activity for CS + LS and PE + LS or to the control level for CS + LE and PE + LE. For longer LOX-polyphenol interaction times (leucocytes cells initially incubated with CS and PE for 3 h followed by the addition of LS and LE and further incubation for total of LOX-polyphenol interaction time of 21 h (*24 *hrs* for 3 *hrs)) antioxidant activities were detected for CS + LE and PE + LS, whereas prooxidant tendency was observed for PE + LE and CS + LS. The activities for the later reaction mix were slightly higher than those observed for 3 h exposure experiments.

### 3.3. Interactions between Lipoxygenase (LS or LE) and Polyphenols (CS and PE) on* E. coli* and* B. linens* Cultures

In the experiments involving mixing CS or PE with LS or LE prior to the addition to* E. coli* and* B. linens* culture, two points of view were taken into consideration: the formation of enzyme-polyphenol complex that may have influence on bacteria and the inhibition of lipoxygenase by polyphenols, thus reducing LOX prooxidant activity. In addition, LOX oxidative catalytic modification of polyphenols may lead the production of*ο*-quinones and other electrophilic prooxidative by-products [35] which by themselves could be toxic to and/or inhibitory to microorganisms [36]. LOX-polyphenol complexion and LOX inhibition by polyphenols and their influence on* E. coli* and* B. linens* culture were analyzed by treating the cell culture with polyphenols for 3 hours before the addition of LOX and the bacterial growth monitored by OD_630 nm_ after 1 h post-incubation with LOX. Under these conditions, there is initial interaction of polyphenols with bacteria cell culture implying their possible oxidative modification prior to addition of the prooxidant LOX.

Figure 3 shows the bacterial growth monitored after 2, 7, 20, and 26 h for* E. coli* cocultivated with LS, LE, CS, and PE alone or in combinations. The addition of CS was associated with instant cessation of growth in* E. coli* culture.* CS + LS 3 *h also exhibited statistically significant antibacterial activity for up to 20 h, when compared with other experimental treatments. However, no significant effect was observed when CS and LS were mixed and added on the* E. coli* culture (*CS + LS 0 *h).

For CS + LE treatment, there was an intense bacterial growth observed upon addition of LE after 7 h, which however decreased after 20 hours of incubation.* CS + LE 0 *h also improved the bacterial growth but proved to be bactericidal at 26 h, a property observed also for CS treated cells. Similarly, the best antibacterial action for PE + LS was recorded after 26 hours of cocultivation and when PE was added 3 h before the LS (*PE + LS 3 *h) or when the two extracts were mixed prior to being added to the bacterial culture (*PE + LE 0 *h).

Figure 4 presents the bacterial growth measured at 2, 7, 20, and 26 h for* B. linens* cocultivated with LS, LE, CS, and PE alone or in mixtures as described in the legends.* Brevibacterium linens* was chosen in this study for comparative analysis on the influence of the polyphenols between a Gram-positive bacteria and a Gram-negative* E. coli B*_41_. In contrast to varied inhibitory activity observed in* E. coli* cell cultures cocultivated with LS, LE, CS, and PE, no antibacterial or bacteriostatic action was observable in the case of* B. linens *(Figure 4).

Interestingly, significant stimulatory effect on microbial growth was observed for PE + LS and PE + LE treatments, illustrating the potential probacterial activity of the polyphenols and LOX in* B. linens *cultures. These results are consistent with the previous study where CS and PE exhibited different effect on the Gram-negative* E. coli* in comparison to the Gram-positive bacteria* B. linens* [25].

Detailed physicochemical studies suggest that the bactericidal activities of galloylated tea catechins at the cell membrane level may be due to their specific perturbations of the ordered structure of phosphatidylcholine and phosphatidylethanolamine bilayers constituting bacterial cell wall membranes [37, 38]. Differential effects of catechins on bacterial cell walls compared to membranes of human cells may be due to differences in structures of the respective walls (membranes) [39]. The bactericidal action of EGCG (epigallocatechin gallate) may depend on hydrogen peroxide derived from the reaction of EGCG with oxygen (prooxidative activity) [40, 41]. These observations suggest that antimicrobial effects arise from the interactions of catechins with oxygen, genes, cell membranes, and enzymes [39]. Postulated antimicrobials mechanisms for botanicals investigated may be disruption of microbial cell membranes and chelation to essential trace elements such as zinc and iron that the bacteria need for growth [42].

## 4. Conclusions

In our present study, the effect of the different oxidation products of grape seed polyphenols in presence of LOX on leucocyte culture was monitored by the TBARS and MTT assays. The TBARS assay revealed that the action of the tested extracts due to the different molecular interactions proves to be time-dependent; longer incubation time of polyphenols and LOX with leucocyte culture generally displayed ant-lipidic peroxidation effect, probably via the complexing of the two classes of molecules and/or LOX inhibition by either CS or PE. While the presence of LOX (LS and LE) was generally associated with higher lipid peroxidation activity of leukocyte cells that was detectable within 3 h, longer incubation time (24 hrs versus 3 hrs) of the same samples in presence of GSE polyphenols resulted in the decrease of peroxidation. In particular, coincubation with polyphenols alone (CS) and coincubation in combination with LS (CS + LS and PE + LS) for 21 h were most effective in inhibiting lipid peroxidation of the cells. However, longer coincubations (24 h) prior to the addition of the LOX enzymes did not significantly lower the lipid peroxidation activity, indicating that autooxidized CS and PE were not effective inhibitors of LOX activity.

Based on the current findings, it can be concluded that either the unoxidized GSE polyphenols and/or their intermediates after 21 h incubation form a complex with LOX thereby suppressing the LOX-mediated lipid peroxidation. It is also plausible that unoxidized GSE polyphenols may participate in the quenching of the highly oxidative free radical species and hydroperoxides liberated during LOX-catalyzed reactions, thus conferring protection to the cells against lipid peroxidation.

Similar to TBARS assay results, MTT test also revealed that longer coincubation time of cells (>24 h) with polyphenols prior to addition of LS resulted in the highest cytotoxicity observed in leukocyte cells. It is generally believed that longer exposure of the cells to polyphenols may lead to polyphenol oxidation to form toxic by-products. Thus, it can be concluded that higher cytotoxicity observed above could be attributable to the effect of polyphenol oxidative intermediates or byproducts rather polyphenol-lipoxygenase interaction. Comparatively, this effect was more pronounced for LS than for LE, indicating a weaker oxidative capacity of LE towards leukocyte cells. CS was also found to be slightly cytotoxic; however, the effect was reversible on addition of LS and LE illustrating the possible beneficial action of catechin-lipoxygenase complex formation.

On the study of the effect of potential lipoxygenase-polyphenols complex formation* in vivo* on bacterial culture, longer exposure (up to 26 hrs) of* E. coli* to CS or PE followed by addition of LS for 3 h resulted in bactericidal activity. Thus, different types of cells may account to different intermolecular interactions involving LOX and polyphenols* in vitro*. Furthermore, Gram-negative bacteria* E. coli* was affected differently from positive* B. linens *in presence of LOX and GSE polyphenols.

In conclusion, our studies have highlighted the beneficial effect of lipoxygenase-polyphenols complex formation in the protection of leucocytes against LOX-mediated lipid peroxidation and cytotoxicity on as wells as imparting antibacterial and probacterial activities on* E. coli* and* B. linens*, respectively. Grape seeds represent an important source of health promoting polyphenols, and thus cost effective technologies will be important in the future for their large scale processing to tap into the high polyphenol content with the beneficial effects on human health.
